# Method for the Characterization of Extreme-Ultraviolet Photoresist Outgassing

**DOI:** 10.6028/jres.114.011

**Published:** 2009-06-01

**Authors:** Charles Tarrio

**Affiliations:** Electron and Optical Physics Division, National Institute of Standards and Technology, Gaithersburg, MD 20899

**Keywords:** contamination, extreme ultraviolet, outgassing, photoresist, vacuum

## Abstract

Outgassing from photoresists illuminated by extreme ultraviolet radiation can lead to degradation of the very expensive multilayer-coated optics in an extreme ultraviolet stepper. Reliable quantification of the various organic molecules outgassed by photoresists has been a challenging goal. We have designed a compact system for this measurement. In the first step, the total number of molecules emitted by the photoresist is measured using a pressure-rise method in a closed vacuum chamber, with the pressure measured by mechanical means using a capacitance displacement gauge. To provide identification and relative abundances, the outgassed molecules are then collected in an evacuated trap cooled by liquid nitrogen for subsequent analysis by gas chromatography with mass spectrometry. We will discuss the design and performance of the system.

## 1. Introduction

Damage to extreme ultraviolet (EUV) and x-ray mirror optics due to carbon deposition in vacuum systems is a well-known effect [1]. It is believed that organic molecules adsorb on the mirror and are then cracked by the impinging photons or resulting secondary electrons to become an amorphous graphitic carbon layer. The carbon layer degrades the optical performance of optics in the EUV region due both to absorption and increasing surface roughness of the layer. This is an especial problem in the water window from 2.5 nm to 4.5 nm wavelength where carbon is strongly absorbing. At synchrotron laboratories the beamlines are often equipped with devices to enable cleaning of the optical surfaces with oxygen plasmas [2]. This works well for mirrors coated with noble metals, but can damage more reactive surfaces.

The quest for a new generation micro-lithography to keep pace with Moore’s Law [3], which states that the number of transistors on a chip doubles roughly every two years, has led to the development of EUV lithography (EUVL) [4] and an increased interest in EUV multilayer optics. The mirrors used in EUVL are coated with multilayer stacks of about 50 Mo/Si bilayers [5] usually capped with an oxidation resistant layer of Ru or TiO_2_ [6]. At normal incidence these optics reflect up to 70 % of the incident EUV light in a narrow spectral band thus allowing excellent-quality image formation. Any small loss of reflectivity due to the carbonization of the surface, however, can dramatically reduce the stepper throughput since a typical stepper design has of order 10 mirrors in the optical train.

Optics damage can come from two sources: oxidation, which is likely irreversible and arises from water vapor; or carbonization, which is potentially reversible and may arise from outgassing of the photoresists during illumination. Oxidation-resistant multilayers have been developed, however outgassing from the photoresist is important enough that there is an effort to develop a uniform specification of what is allowable. Early results of measurements of outgassing during EUV illumination have shown orders of magnitude differences among different laboratories, and great variation even within the same laboratory [7]. These measurements relied either on ionization gauges to measure pressure rise or trapping of gases using desorption tubes with subsequent counting of molecules using gas chromatography with mass spectrometry (GCMS).

Given the wide disparity in results, we have decided upon a new approach. To determine the total number of molecules outgassed by the resist, we measure the total pressure rise using mechanical gauges that measure pressure with little or no dependence on gas composition. To determine the composition of the outgassed molecules, we use a liquid nitrogen-cooled trap and subsequent GCMS analysis.

One gauge for measurement of pressure with no gas dependence is a capacitance displacement gauge (CDG), a version of which is available for measurement of pressures between about 10^–5^ hPa and 0.1 hPa. A second type of mechanical gauge is the spinning rotor gauge (SRG), which is sensitive an order of magnitude above and below the CDG. The SRG measures the decay in rotation rate of a small ball, or rotor, thus its response is proportional to the square root of the molecular weight. A typical number of outgassed molecules per unit area from an EUV photoresist is of order 10^14^ cm^–2^, which leads to a pressure rise of about 0.3 hPa, assuming a 100 mm diameter wafer and a 1 L chamber volume. In order to maximize the pressure rise, we must minimize the volume into which the sample is allowed to outgas.

**Fig. 1:**
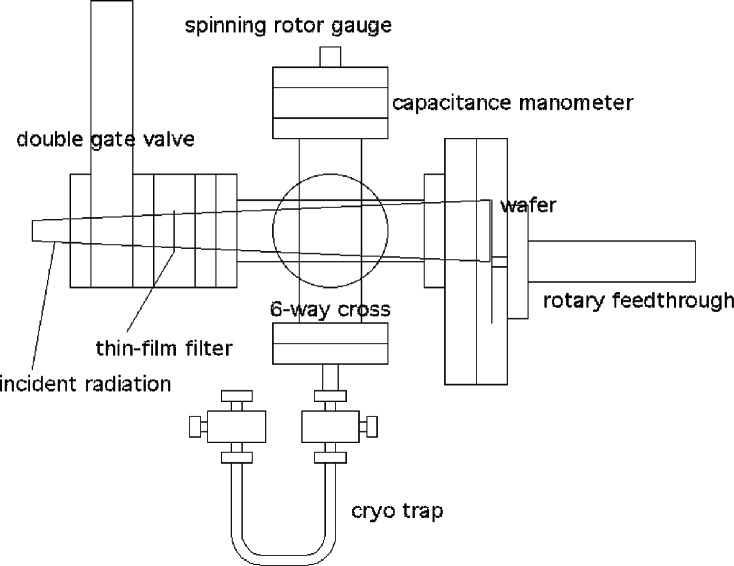
Schematic diagram of our resist-outgassing apparatus.

Our final design is shown in Fig. 1. Not shown are the Synchrotron Ultraviolet Radiation Facility (SURF III) storage ring [8] and a 75 mm diameter MoSi multilayer collecting mirror with 1.5 m radius of curvature. The mirror accepts about 16.6 mrad of the horizontal emission of the SURF III output and the full 3 mrad of the vertical emission and also serves as a wavelength selector. The mirror is 4.5 m from the tangent point of the electron beam and focuses the radiation 90 cm downstream. The entrance aperture of the outgassing chamber is 25 cm from the focus. The radiation cone is 83 mrad horizontally and 15 mrad vertically. In order to illuminate the wafer uniformly and minimize the chamber volume, the entrance aperture is triangular, making a triangular footprint on the wafer, which is mounted off-center and continuously rotated. The aperture has a mesh-backed 0.3 μm Zr filter, which provides further spectral filtering while also providing a vacuum seal. About 90 % of the radiation incident on the sample is in a 1 nm band around 13.3 nm. The vacuum seal is necessary both to define the outgassed volume and to prevent upstreaming of the damaging outgas components to the multilayer mirror and into the storage ring. The filter is captured in the gate of a double gate valve and can hold off of order 1 Pa pressure without breaking. The second gate in the gate valve allows venting to atmosphere while minimizing the path length to the sample, and thus the outgassing volume. To uniformly illuminate a 76 mm diameter wafer, the main chamber is a 6-way, 3.5 cm diameter, stainless steel cross. An off-center 15 cm flange mates to the sample, which sits in a milled-out flange and is rotated by a motorized bellows-sealed feedthrough. The other ports of the cross go to pumping and gauging as well as a small tube, which allows trapping of outgas components for subsequent chemical analysis.

**Fig. 2:**
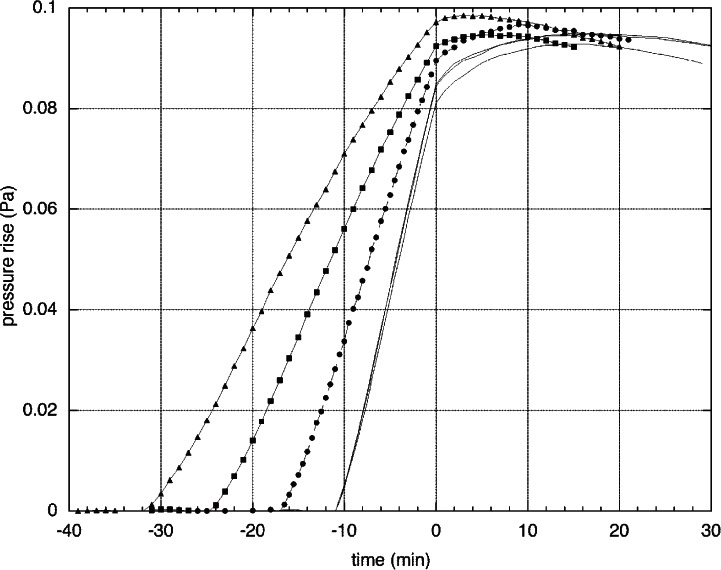
Pressure rise for six different runs on the same photoresist. The origin of the time scale is the time when the exposure is finished. Three runs are at 1.2 mW/cm^2^ intensity (three overlapping curves), the others at 0.8 mW/cm^2^ (circles), 0.6 mW/cm^2^ (diamonds) and 0.4 mW/cm^2^ (triangles).

To make a measurement, all pumping is valved off. Then the background pressure rise is measured, usually for 10 min. Once a linear background is obtained, the sample is illuminated until 2.5× the dose-to-clear. (The dose-to-clear is determined by making several static exposures of different duration on a wafer to determine where the resist is fully cleared.) The pressure is monitored during illumination and for some period after. Figure 2 shows the results of several runs on one photoresist. In each run, a linear background was established for 10 min before the run and subtracted from the results. Three runs were made at full beam current, corresponding to about 1.2 mW/cm^2^ intensity. The other three were made at lower intensities of 0.8 mW/cm^2^, 0.6 mW/cm^2^, and 0.4 mW/cm^2^. The time scale is adjusted so that 0 corresponds to the time at which the exposure was finished. Note that the subsequent pressure rise is not linear, and it also depends on the illumination intensity. In this case, determining the total rise 10 min to 20 min after stopping the exposure leads to relative uncertainties in the analysis method of only 3 % (standard uncertainty with coverage factor *k* = 1), which is small compared to the other uncertainties in the measurement. This persistence in the out-gassing varies from resist to resist, with some displaying virtually no continued pressure rise and some displaying a pressure rise continuing for as much as an hour after stopping illumination.

Photoresist outgassing is dependent on the film thickness and total dose. The dominant uncertainties in our measurements are thickness of the resist and dose to clear, both of which contribute about 10 % to our uncertainty. Other components include the analysis uncertainty discussed above, at 3 %, outgassing volume, also about 3 %, background determination and pressure measurement, both about 1 %. This leads to a root-sum-square uncertainty of 15 % in the measurement of the total molecules outgassed.

**Fig. 3:**
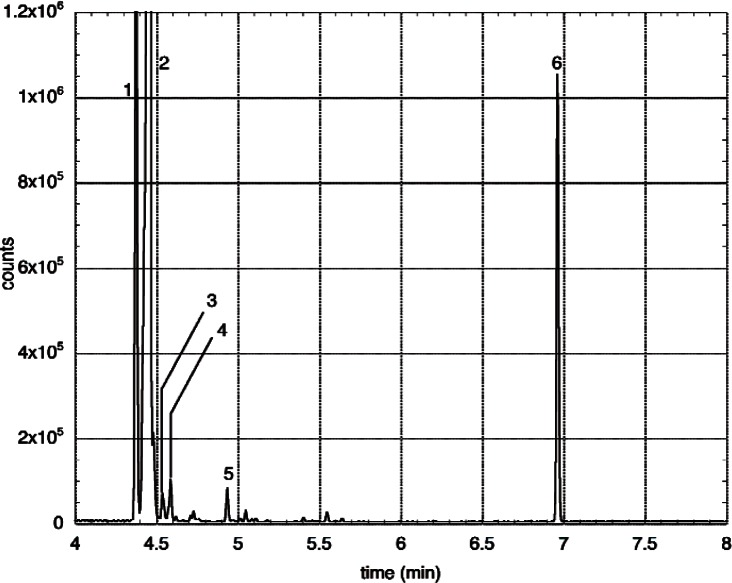
GCMS run from the outgassing of a second photoresist. The peaks are 1: CO_2_; 2: isobutene with isobutane and water as shoulders; 3: acetone; 4: iodomethane; 5: benzene; 6: tert-butylbenzene.

After the total outgassing has been measured, a small tube is cooled to liquid-nitrogen temperature and a valve to the chamber opened to freeze out the outgas product. About 80 % of the gas in the chamber is trapped this way. We have experimented trapping pure gases and have found good efficiency for gases with melting temperatures higher than that of CO. This tube is then attached to the inlet of a GCMS instrument. The injection is made by switching a six-way valve so that helium flows through the sample tube, which is heated to 110 °C, for 2 min, then the valve is switched back so that the helium is flowed directly onto the GC column. The column is cooled with a cryo focus, which cools about 3 mm of the column to near liquid nitrogen temperature. This allows trapping of any species collected by our trap and also sharpens up peaks in the chromatogram. Figure 3 shows a chromatogram from a second photoresist we’ve tested. The first peak is CO_2_, which is offscale at about 2 × 10^6^ counts. The second is dominated by isobutene (offscale at 10^7^ counts), however, by analyzing the mass spectra, smaller peaks are revealed: isobutane just before the main peak, and water just after the main peak, at a level approximately the same as the background run made immediately before the data run. Four more peaks are clearly identified, in ascending order: acetone, iodomethane, benzene, and tert-butylbenzene.

The measurement of photoresist outgassing using species-independent vacuum measurement is a considerable improvement over previous techniques. However caution must be exercised in interpreting the results due to possible slow processes in some resists. Outgassed molecules are efficiently trapped using a liquid-nitrogen cooled tube. The tube has a small enough volume to allow efficient injection into a GCMS instrument, which allows a sensitive determination of the chemical makeup of the outgassed species. These species will then be studied in an environmental exposure chamber to determine which are the most damaging chemicals, allowing resist suppliers to design resists without the most damaging species.
